# Ketoprofen-loaded quatsomes as a smart repurposed antifungal therapy for vaginal infections: formulation, characterization, and microbiological evaluation

**DOI:** 10.3389/fphar.2026.1767624

**Published:** 2026-02-27

**Authors:** Alaa Emad Eldeeb, Diana E. Aziz, Mariam Hassan, Osama Saher, Sadek Ahmed

**Affiliations:** 1 Department of Pharmaceutics and Industrial Pharmacy, Faculty of Pharmacy, Cairo University, Cairo, Egypt; 2 Department of Microbiology and Immunology, Faculty of Pharmacy Cairo University, Cairo, Egypt; 3 Department of Microbiology and Immunology, Faculty of Pharmacy, Galala University, New Galala, Egypt; 4 Department of Laboratory Medicine, Karolinska Institute, Stockholm, Sweden; 5 Department of Cellular Therapy and Allogeneic Stem Cell Transplantation (CAST), Karolinska University Hospital Huddinge and Karolinska Comprehensive Cancer Center, Stockholm, Sweden

**Keywords:** invivo microbiological model, ketoprofen, mixed factorial design, quatsomes, repurposed drug, vaginal candidiasis

## Abstract

**Introduction:**

Vulvovaginal candidiasis (VVC) is one of the most common fungal infections requiring more effective and patient-friendly therapies. This study introduces repurposed Ketoprofen (KPN) Quatsomes (QS) as a novel nano-platform for localized antifungal treatment.

**Methods:**

KPN-QS were prepared using quaternary ammonium surfactants and cholesterol via probe sonication and optimized through a 3^1^ × 2^2^ mixed factorial design using Design-Expert^®^ software. The effects of quaternary ammonium surfactant type (factor A), amount of vesicle-forming materials (factor B), and cholesterol-to-surfactant ratio (factor C) were evaluated to maximize entrapment efficiency and zeta potential, while minimizing particle size.

**Results:**

The optimized QS exhibited spherical nano-sized vesicles (113.7 nm) with high entrapment efficiency (96.8%) and strong positive zeta potential (72.5 mV), ensuring stability and enhanced mucosal adhesion. TEM confirmed the spherical morphology, and *in vitro* release showed biphasic behaviour with 86.5% release after 8 h, alongside excellent storage stability. Repurposing KPN as an antifungal agent significantly enhanced both *in vitro* and *in vivo* microbiological efficacy. The formulation displayed promising MIC values against *Candida albicans* and markedly improved antifungal performance *in vivo* VVC model. The KPN-QS group exhibited 4.807 and 2.941 log reductions in fungal count compared to the negative control and KPN suspension, respectively, with complete eradication in three rats after 72 h. Histopathological analysis confirmed the safety of QS on vaginal mucosa.

**Conclusion:**

Collectively, repurposed KPN-QS constitute a stable, biocompatible nanocarrier for targeted vaginal delivery, demonstrating superior antifungal activity and therapeutic potential in VVC.

## Graphical Abstract

**Figure:**
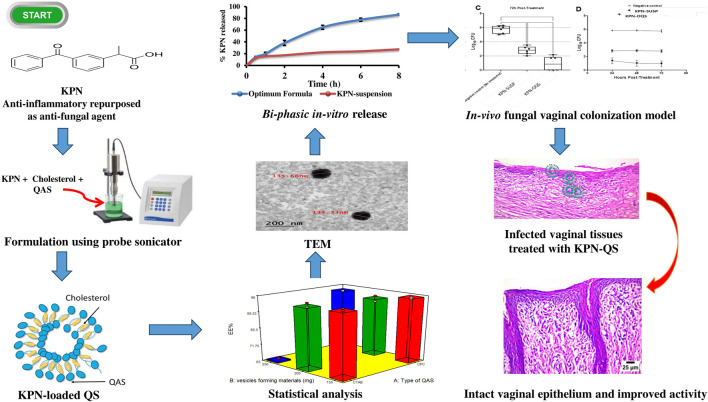

## Highlights

- Repurposed Ketoprofen was successfully formulated into Quatsomes (KPN-QS) as a novel antifungal nano-platform for vulvovaginal candidiasis.
- Mixed factorial design (3^1^ × 2^2^) enabled optimization of key parameters: EE% of 96.8%, PS of 113.7 nm and ZP of 72.5 mV.
- TEM confirmed uniform spherical vesicles, while *in vitro* studies revealed biphasic sustained release (86.5% after 8 h) and excellent storage stability.
- Repurposing Ketoprofen significantly enhanced antifungal activity, demonstrating promising *in vitro* MIC values against *Candida albicans.*
- *In vivo* evaluation showed superior efficacy, achieving 4.807 and 2.941 log reductions in fungal count vs. negative control and KPN suspension.
- Histopathological analysis verified mucosal safety and biocompatibility, supporting localized vaginal delivery with improved therapeutic outcomes.

## 1 Introduction

Vulvovaginal candidiasis (VVC) is a localized infection affecting the mucosal surfaces of the vagina and the vulva, caused by *Candida* species (J et al., 2024; Ahmed et al., 2026a; Minooeianhaghighi et al., 2020). *Candida albicans (C. albicans)* is responsible for approximately 80% of these infections and exerts its pathogenicity through a combination of virulence factors that promote colonization, tissue invasion, and immune evasion (Czechowicz et al., 2022; Goncalves et al., 2016). The fungus expresses cell-wall adhesins that mediate strong binding to vaginal epithelial cells and can undergo a morphological switch from yeast to hyphal forms, facilitating deeper tissue penetration. During hyphal growth, *C. albicans* secretes candidalysin, a cytolytic peptide toxin that damages epithelial cells and triggers strong proinflammatory responses (Moyes et al., 2016). It also produces hydrolytic enzymes (such as proteases and phospholipases) that degrade host barriers. The organism also forms resilient biofilms that enhance persistence and resistance particularly under conditions of hormonal or microbial flora imbalance. Collectively, these factors contribute to the high virulence of *C. albicans* as a significant vaginal pathogen (Lopes and Lionakis, 2022; Ahmed et al., 2025a).

Up to 80% of women experience VVC at least once in their lifetime, with recurrent episodes in 40%–50% of patients (Salah et al., 2018; Albash et al., 2020). Vaginal candidiasis causes significant discomfort to women, including itching, burning, and abnormal discharge (Fukazawa et al., 2019). Moreover, if left untreated, it may lead to serious secondary bacterial infections or even systemic infections, particularly in elderly and immunocompromised women (Li et al., 2022; Zhou et al., 2009). Notably, the incidence of vaginal candidiasis increases during pregnancy due to hormonal and immunological changes. Inadequate management of these infections may result in serious complications, such as preterm labor, low birth weight, and an increased risk of neonatal invasive candidiasis (Goncalves et al., 2016; Disha and Haque, 2022).

Conventional vaginal drug delivery systems, such as gels, creams, pessaries, and tablets, are widely used for local treatment of infections and hormone therapy. However, these formulations suffer from several limitations that reduce their therapeutic efficacy and patient compliance (Albash et al., 2020). These challenges include (i) the short residence time brought on the vaginal fluidsʼ self-cleansing action, gravity, and mucociliary clearance, (ii) uneven distribution of conventional dosage forms, and (iii) limited penetration through the vaginal mucosa. Moreover, conventional antifungal therapies (e.g., azoles and polyenes) are increasingly challenged by drug resistance, limited activity against biofilm-forming *Candida* species, and frequent recurrence rates (Sobel et al., 2023; Sasi et al., 2025).

Nanotechnology-based vaginal drug delivery systems offer a promising approach to overcome the limitations of conventional formulations, including poor vaginal retention, limited mucosal penetration, and the development of drug resistance. Many studies on liposomes (Czech et al., 2019; Refai et al., 2017), polymeric nanoparticles (Leyva-Gomez et al., 2018; Teama et al., 2025), nanoemulsions (Basu et al., 2025), transferosomes (Hady et al., 2022), flexosomes (Ahmed et al., 2025a) have demonstrated the superiority of nanosystems over traditional formulations. These systems enhance mucoadhesion, improve drug solubility and stability, and provide a controlled drug release. Additionally, they facilitate deeper mucosal penetration and prolong residence time within the vaginal environment, making them highly effective for treating vaginal infections and delivering hormonal or anticancer agents (Chindamo et al., 2021; Negi et al., 2023). Consequently, there is a critical need for alternative nanocarrier-based delivery systems that enhance vaginal penetration, prolong mucosal residence time, and enable the incorporation of novel antifungal agents to overcome the increasing resistance associated with conventional therapies.

Drug repurposing has emerged as an efficient and cost-effective strategy for accelerating drug development by discovering new clinical use for established drugs. This approach bypasses many time-consuming stages of drug development, thereby reducing development costs, the number of regulatory approvals required, and minimizing the attrition rates associated with conventional drug development (Elmahboub et al., 2025; Hua et al., 2022).

Ketoprofen (KPN), a widely used non-steroidal anti-inflammatory (NSAID), has recently drawn interest for its potential antifungal activity, offering a novel repurposing opportunity. Unlike conventional antifungals that target ergosterol biosynthesis or fungal cell walls, KPN exerts antifungal effects by inhibiting fungal cyclooxygenase-like pathways and interrupting prostaglandin-mediated virulence mechanisms in *Candida* species. Studies have shown that KPN can diminish the key factors involved in the persistence and recurrence of *Candida* infections (e.g., hyphal formation, biofilm development, and fungal adhesion) (Vanic et al., 2021).

Quatsomes (QS) are a novel class of lipid-based nanovesicles formed by the self-assembly of cholesterol and quaternary ammonium surfactant (QAS) in aqueous media. These nanostructures have attracted considerable attention for their versatility in various biomedical applications, including bioimaging and drug delivery (Vargas-Nadal et al., 2020). Compared to conventional liposomes, quatsomes exhibit superior colloidal stability, size homogeneity, structural uniformity, and high drug encapsulation efficiency, making them highly promising carriers for controlled drug delivery (Battista et al., 2022). Moreover, the incorporation of QAS into the vesicle membrane imparts antimicrobial activity, further enhancing their therapeutic potential, particularly in the treatment of infections (Hassan et al., 2020).

In this context, the present study aims to develop and optimize quatsomes drug delivery system for the vaginal delivery of KPN, offering a promising alternative for the treatment of VVC. Quatsomes were employed to enhance drug solubility, mucosal penetration, and retention; therefore, they provide enhanced therapeutic efficacy at the site of infection. To achieve this, a 3^1^.2^2^ combined full factorial was employed to evaluate the influence of the formulation variables on the desired responses. Following comprehensive *in vitro* characterization and statistical optimization using Design-Expert^®^ software, the formulations were evaluated *in vivo* in a rabbit model of vaginal candidiasis to assess therapeutic performance. The optimized formula proved the potential of KPN-QS in managing resistant or recurrent mucosal fungal infections as the first formulated nanovesicles of KPN for fungal infections.

## 2 Materials and methods

### 2.1 Materials

Ketoprofen (KPN) was supplied as a gift from the Egyptian International Pharmaceutical Industries (EIPICO; Cairo, Egypt). Cholesterol, Cetyltrimethylammonium bromide (CTAB) and cetyl pyridinium chloride (CPC) and dialysis membrane (molecular weight cut-off of 12,000–14,000 g/mol) were purchased from Sigma Aldrich (Darmstadt, Germany). The remaining other reagents were all of analytical grade and used as supplied. The used was deionized.

### 2.2 Animals

Healthy adult Wistar rats (150 ± 7 gm) were selected for the *in vivo* evaluations related to vaginal drug delivery. The animals were housed individually in sanitized, well-ventilated cages under controlled laboratory conditions (25 °C ± 2 °C) with a 12-h light/dark cycle. They were maintained on a standard commercial diet and provided with unrestricted access to clean drinking water. Prior to experimentation, all animals underwent thorough veterinary examination to confirm their general health and exclude any signs of vaginal infection, inflammation, or systemic abnormalities. All animal-handling procedures and experimental protocols were conducted following internationally accepted ethical standards. The Research Ethics Committee for Experimental and Clinical Studies, Faculty of Pharmacy, Cairo University, examined and approved the study protocol (Approval No. MI 3893). All procedures followed the ARRIVE guidelines to ensure experimental reliability, ethical integrity, and the welfare of the animals.

### 2.3 Statistical design and factorial analysis

A mixed-level full factorial design (3^1^ × 2^2^) was systematically employed using Design-Expert^®^ software (Stat-Ease Inc., Minneapolis, MN, United States) to optimize the formulation of Ketoprofen-loaded quatsomes (KPN-QS) for enhanced vaginal antifungal therapy. This statistical approach was selected for its efficiency in simultaneously exploring multiple formulation variables, identifying significant factor interactions, and minimizing the number of experimental runs required to develop a robust model (Farag et al., 2025). Three critical independent variables were investigated based on preliminary trials: the type of quaternary ammonium surfactant (QAS) as either CTAB or CPC (factor A); the total amount of vesicle-forming materials (150, 200, or 250 mg) (factor B); and the cholesterol-to-QAS molar ratio (1:1 or 3:1) (factor C). These formulation factors were selected for their expected impact on the structural integrity, surface charge, and encapsulation performance of the developed vesicles. To assess the overall formulation quality and guide optimization, three key dependent responses were evaluated: entrapment efficiency (EE%, Y_1_) to be maximized, particle size (PS, Y_2_) to be minimized, and zeta potential (ZP, Y_3_) to be maximized (Ahmed et al., 2026b). These physicochemical attributes were chosen to ensure stable nano-sized carriers with high drug payload, enhanced mucosal adherence, and improved local retention (Ahmed et al., 2025b; El Hassab et al., 2025). The detailed factor levels and constraints applied in the factorial design are listed in Table 1, whereas the full composition of the prepared formulations along with the observed experimental outcomes are summarized in Table 2. Statistical analyses were performed at a significance level of *p* < 0.05 to identify the most influential parameters and define the optimal formulation (Younes et al., 2024).

**TABLE 1:** Mixed-level 3^1^2^2^ full factorial design demonstrates factorial levels and target constraints.

Factors (independent variables)	Level		
	−1	0	+1
A: Type of QAS B: Amount of vesicles forming materials (mg) C: Ratio of cholesterol and QAS	CTAB 150 1:1	- 200 -	CPC 250 3:1
Responses (dependent variables)	Desirability constraints		
Y_1_: EE % Y_2_: PS (nm) Y_3_: ZP (absolute value) (mV)	Maximize Minimize Maximize		

Abbreviations: CPC, cetyl pyridinium chloride; CTAB, cetyltrimethylammonium bromide; EE %, percent entrapment efficiency; PS, particle size; QAS, quaternary ammonium surfactant; ZP, zeta potential.

**TABLE 2:** Composition and characterization of the prepared KPN-QS^a^.

Formulation code^b^	A:type of QAS	B: amount of vesicles forming materials (mg)	C: ratio of cholesterol and QACs	EE %	PS (nm)	PDI	ZP (mV)
QS_1_	CTAB	150	1:1	96.9 ± 0.6	108.7 ± 2.5	0.23 ± 0.01	60.3 ± 5.4
QS_2_	CPC	150	1:1	96.8 ± 0.6	113.7 ± 2.3	0.35 ± 0.01	72.5 ± 3.3
QS_3_	CTAB	200	1:1	96.0 ± 1.4	124.2 ± 5	0.42 ± 0.03	36.4 ± 0.6
QS_4_	CPC	200	1:1	93.7 ± 2.1	110 ± 2.2	0.35 ± 0.01	67.3 ± 2.2
QS_5_	CTAB	250	1:1	64.2 ± 0.7	130.2 ± 35.4	0.26 ± 0.01	79.4 ± 3.4
QS_6_	CPC	250	1:1	95.5 ± 0.2	106.6 ± 0.7	0.36 ± 0.04	66.1 ± 1.6
QS_7_	CTAB	150	3:1	96.8 ± 2.3	132.5 ± 1.6	0.24 ± 0.01	67.3 ± 2.1
QS_8_	CPC	150	3:1	41.2 ± 0.5	123 ± 3.5	0.31 ± 0.01	40.8 ± 6.9
QS_9_	CTAB	200	3:1	48.2 ± 1.7	328 ± 13.4	0.27 ± 0.02	43.8 ± 2.6
QS_10_	CPC	200	3:1	68.0 ± 1.1	135.3 ± 5.7	0.23 ± 0.01	70.5 ± 0.7
QS_11_	CTAB	250	3:1	24.4 ± 0.4	132.1 ± 39.4	0.25 ± 0.02	70.3 ± 6.3
QS_12_	CPC	250	3:1	94.8 ± 0.6	133.8 ± 0.9	0.25 ± 0.01	66.4 ± 0.9

^a^ Trials are listed in standard order.

^b^ All the prepared formulae contained 5 mg/mL KPN.

Data are presented as mean ± SD.

Abbreviations: CPC, cetyl pyridinium chloride; CTAB, cetyltrimethylammonium bromide; EE %, percent entrapment efficiency; PDI, poly-dispersity index; PS, particle size; QAS, quaternary ammonium surfactant; QS, Quatsomes ZP, zeta potential.

### 2.4 Preparation of ketoprofen quatsomes (KPN-QS)

The sonication technique was preferred among various preparation methods due to its simplicity, widespread applicability, and for eliminating the need for organic solvents during the formulation process (Shohin et al., 2012). Quatsomes were prepared by adding 50 mg KPN with accurately weighed amounts of QAS (CTAB or CPC) and cholesterol in 10 mL distilled water, the mixture was placed in an ice bath and sonicated for 30 min at 20/5 s on/off cycle and 40% amplitude using a QSonica probe sonicator (Newton, CT, United States) (Schroeder et al., 2009). Next, a magnetic stirrer operating at 400 rpm was used to agitate the dispersion for 30 min at room temperature (WiseStir, Daihan Scientific C. o., Ltd., Korea). Finally, the prepared formulae were kept in the refrigerator at 4 °C till characterization (Ahmed et al., 2025c).

### 2.5 *In vitro* evaluation of the prepared quatsomes

#### 2.5.1 Entrapment efficiency assessment (EE%)

The EE% of KPN in the prepared quatsomes was determined by separating the unentrapped drug through filtration using Whatman filter paper (grade No. 1, pore size 11 μm) (El Hassab et al., 2025). An aliquot of 1 mL from the filtrate was subsequently sonicated in methanol to ensure complete drug release, and the concentration of KPN was quantified spectrophotometrically at λ_max_ = 257 nm (*R* ^2^ = 0.995). Measurements were conducted in duplicate, and the results were expressed as the mean ± standard deviation (SD). The EE% was calculated according to the following equation (El-Shahed et al., 2024; Taw et al., 2025):

$$EE\%=\frac{encapsulated\;amount\;of\;KPN}{Total\;amount\;of\;KPN}\times 100$$ (1)

#### 2.5.2 Particle size, poly-dispersity index, and zeta potential

The physicochemical characterization of the prepared KPN-QS was conducted to evaluate their colloidal performance and predict formulation stability and vaginal adhesion potential. Key parameters including particle size (PS), poly-dispersity index (PDI), and zeta potential (ZP) were analysed, as these serve as critical indicators of nanosystem homogeneity, electrostatic stability, and mucoadhesive potential (Ahmed et al., 2025d). Measurements were performed using a Zetasizer Nano ZS (Malvern Instruments Ltd., Worcestershire, United Kingdom) operated at 25 °C and a fixed backscattering angle of 173° (Ahmed et al., 2025e). Prior to analysis, freshly prepared formulations were diluted (1:10 v/v) with distilled water until a faint opalescence as obtained, ensuring suitable particle dispersion and minimizing multiple scattering or vesicle aggregation. Each diluted sample was gently vortexed to achieve homogeneity before measurement (Fahmy et al., 2025; Saher et al., 2019). The PDI values were employed to assess the uniformity of the vesicular population, while ZP values were determined through electrophoretic mobility analysis, reflecting the surface charge intensity and predicting colloidal stability (Ahmed et al., 2025f). All measurements were performed in triplicate, and the mean ± standard deviation (SD) was used express the results to ensure reproducibility and analytical reliability (Farag et al., 2023; Saher et al., 2023).

### 2.6 Statistical optimization of the prepared quatsomes

A numerical desirability function approach was applied, allowing simultaneous consideration of all critical responses and integration of their outcomes into a single composite index ranging from 0 (least desirable) to 1 (most desirable) (Ahmed et al., 2025g). The desirability-based optimization targeted formulations with the highest EE% and ZP values, coupled with the smallest PS, as these attributes collectively contribute to improved bio-adhesion, penetration, and sustained antifungal activity (Albash et al., 2025a). Based on statistical analysis and analysis of variance (ANOVA) results, the formulation exhibiting the highest overall desirability value was identified as the optimized KPN-QS and subsequently prepared for confirmatory characterization and microbiological evaluation. The close agreement between predicted and experimental responses (with deviations <5%) validated the robustness and predictive accuracy of the employed statistical model, confirming its reliability for guiding nanosystem development (Ahmed et al., 2024a).

$$\%\text{ Deviation}=\left(\left|\text{Predicted value}-\text{Observed value}\right|/\left(\text{Observed value}\right)\right)\times 100$$ (2)

### 2.7 *In vitro* analysis of the optimized QS

#### 2.7.1 *In vitro* drug release

The release of KPN from KPN-OQ was evaluated using dialysis bag diffusion technique (Ahmed et al., 2025h). 0.5 mL of the formulation (equivalent to 2.5 mg KPN) was added to a dialysis bag (molecular weight cut-off: 12,000–14,000 Da) that had been pre-soaked overnight in release medium. Both ends of the bag were firmly sealed to prevent any leakage during the experiment (Ragheb et al., 2025). Each filled dialysis bag was then placed into a glass bottle containing 50 mL of phosphate buffer saline, pH 4.5 containing 5% methanol kept under continuous stirring set at 100 rpm and 37 °C ± 0.5 °C (Hot plate magnetic stirrer, WiseStir, Daihan Scientific C. o., Ltd., Korea) (Ahmed et al., 2025a). To maintain sink conditions, 3 mL samples were taken out at predefined intervals (0.5, 1, 2, 4, 6, and 8 h) and promptly replaced with an equivalent volume of fresh buffer (Ahmed and Badr-Eldin, 2019). The collected samples were analysed spectrophotometrically at 257 nm to determine the concentration of KPN. A release study of KPN-SUSP in a buffer was conducted under identical conditions to verify the appropriateness of the dialysis membrane used and to compare the release profile of the suspension with the prepared quatsomes. The cumulative percentage of KPN released was computed at each time point and plotted against time (Ahmed et al., 2020). To better understand the mechanism governing KPN release from the optimized formulation, the obtained release data were mathematically analyzed using multiple kinetic models, including zero-order, first-order, Higuchi diffusion, and Korsmeyer–Peppas equations. These models were applied to determine the predominant release model. The correlation coefficient (*R* ^2^) values for each model were calculated, and the model exhibiting the highest *R* ^2^ was considered the most appropriate descriptor of the release behavior (Ahmed et al., 2024b; Abdelhakeem et al., 2025).

#### 2.7.2 Transmission electron microscope

A sample of KPN-OQ was placed onto a copper grid coated with carbon after being suitably diluted with distilled water. After removing the excess dispersion with filter paper, the sample was allowed to air dry at room temperature for 10 minutes (Albash et al., 2025b). It was then visualized using transmission electron microscope (TEM) (Hitachi HF-2000, Tokyo, Japan) operated at an accelerating voltage of 80 kV to examine the morphology of the selected formula (Elmahboub et al., 2024; Ahmed et al., 2023).

#### 2.7.3 Effect of short term storage stability

Particle growth, drug leakage, and other possible alterations were tracked to evaluate the stability of KPN-OQS. KPN-OQS was refrigerated for 3 months, and then its PS, PDI, EE%, ZP, and drug release profile were compared to freshly prepared samples (Ahmed et al., 2022). With SPSS® software (version 22.0; IBM Corp., Armonk, NY, United States), statistical analysis was performed using the paired Student’s t-test, with p < 0.05 deemed significant (Fahmy et al., 2026).

### 2.8 Microbiological and *in vivo* analysis

#### 2.8.1 *In vitro* antifungal activity

The antifungal activity of KPN against *C. albicans* ATCC10231 was evaluated (Al-mahallawi et al., 2021). The microdilution method was used to estimate the minimum inhibitory concentration (MIC) in accordance with the Clinical and Laboratory Standards Institute’s recommendations as previously described (El-Hema et al., 2025; Ismail et al., 2021). Briefly, the tested KPN formulations were serially diluted in Sabouraud Dextrose Broth (SDB) to obtain a range of concentrations (12.5–0.0224 mg/mL). A standardized fungal inoculum (10^5^–10^6^ CFU/mL) was prepared and added to each well of sterile 96-well microtiter plates. Microplates were incubated at 30 °C for 24–48 h, and fungal growth was assessed visually and spectrophotometrically by measuring optical density at 600 nm using a microplate reader (Biotek Synergy 2, SLFA model, United States). The minimum inhibitory concentration (MIC) was defined as the lowest concentration of the tested formulation that resulted in complete inhibition of visible fungal growth compared to the growth control. All experiments were performed independently in triplicate, and results were expressed as mean ± standard deviation.

#### 2.8.2 *In vivo* fungal vaginal colonization model

KPN-OQS was tested *in vivo* to validate its antifungal activity in a vaginal drug delivery system. Female Wistar rats (150 ± 7 gm) were used in a murine *Candida* vaginal colonization model as described before (Eltabeeb et al., 2024; Ali et al., 2023). Estradiol (0.5 mg) was intraperitoneal injected 24 h before starting the fungal colonization. Dexamethasone (0.4 mg) was also injected intraperitoneally once daily, starting 24 h before colonization and continuing for three consecutive days during the colonization process. Fungal colonization was established by vaginally inoculating the rats with a suspension of *C. albicans* ATCC10231 (2-5 × 108 CFU) over a period of four consecutive days. Rats were randomly distributed into three groups (n = 6) and treatments were administered inter-vaginally (250 µL of the corresponding treatment using a soft plastic tip) 24 h post the last inoculation. The group size of six rats per treatment group was determined based on ethical considerations aligned with the 3Rs (Replacement, Reduction, Refinement) principle, aiming to minimize animal use while ensuring scientific validity. One group served as the negative control group and did not receive any treatment. The second group was treated with the KPN-SUSP. The third group was treated with the KPN-OQS. The vaginal lumen samples were collected from each rat using a sterile swab at 24, 48, and 72 h after treatment). The recovered *C. albicans* in the swaps were serially diluted in PBS and spotted on Sabouraud dextrose agar for the viable count as described before (Eltabeeb et al., 2024). The results of the tested groups were analysed and compared.

#### 2.8.3 Histopathological evaluation

Vaginal specimens were excised and promptly fixed in 10% neutral buffered formalin. The samples were then processed using standard histological techniques: trimming, dehydration in ascending grades of ethanol, clearing in xylene and embedding in paraffin wax (Ahmed et al., 2021). Sections of 4–6 μm thickness were obtained using a rotary microtome and subsequently stained with hematoxylin and eosin (H&E) for general histopathological examination, following the method described by Bancroft and Gamble (Ahmed et al., 2025a).

## 3 Results and discussion

### 3.1 Preparation of ketoprofen quatsomes (KPN-QS)

Formulating KPN-QS offers multiple therapeutic advantages for vaginal delivery, including enhanced drug solubility and localized absorption, electrostatic mucoadhesion to the negatively charged vaginal mucosa for prolonged retention, and protection of the drug from the vaginal fluid turnover. Quatsomes were prepared via probe sonication method to enhance the quality of the prepared nanovesicles, as it induces intense cavitation, promotes efficient dispersion of surfactant and cholesterol molecules, facilitates bilayer formation, and breaks down larger aggregates into smaller, more uniform vesicles (Schroeder et al., 2009). This high energy input reduces vesicle size, narrows the PDI, and improves colloidal stability without compromising the bilayer integrity (Ferrer-Tasies et al., 2013).

### 3.2 Analysis of mixed factorial design

To achieve a systematic and efficient optimization of KPN-QS, a mixed-level full factorial design (3^1^ × 2^2^) was employed using Design-Expert^®^ software (Stat-Ease Inc., Minneapolis, United States). This experimental strategy enabled the simultaneous evaluation of the individual and interactive effects of three critical formulation parameters: type of QAS (factor A), amount of vesicle-forming materials (factor B), and cholesterol-to-QAS ratio (factor C). Preliminary screening studies were conducted to establish feasible limits for each factor, ensuring practical formulation ranges (Ahmed et al., 2025b). The selected responses were identified as key quality attributes to guarantee high drug encapsulation, nanoscale uniformity, and colloidal stability. The experimental data were analyzed using polynomial regression and response surface methodology (RSM) to explore the relationships between variables and responses. Regarding the results of the design analysis, It's notable that the predicted *R* ^2^ values closely matched the adjusted *R* ^2^ values for all responses, confirming that the selected model fits the data adequately (Table 3) (Younes et al., 2025).

**TABLE 3:** Output data of the mixed 3^1^ × 2^2^ factorial design implemented for optimization of KPN-QS.

Response	*R* ^2^	Adjusted *R* ^2^	Predicted *R* ^2^	Adequate precision	Significant factors
EE (%)	0.9988	0.9978	0.9954	84.68	A, B, C, AB, BC, ABC
PS (nm)	0.9628	0.9288	0.8514	19.56	A, B, C, AB, AC, BC, ABC
ZP (mV)	0.8807	0.7714	0.7229	8.85	B, AB, ABC

Abbreviations: EE %, percent entrapment efficiency; PS, particle size; ZP, zeta potential.

#### 3.2.1 Analysis of EE%

**FIGURE 1:**
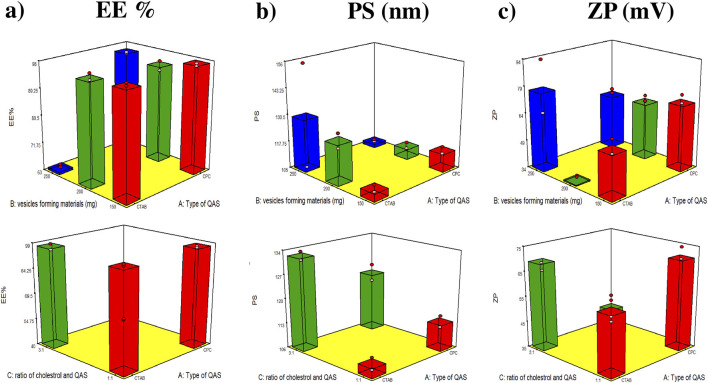
Response 3D plots for the effect of Type of QAS (Factor A), Amount of vesicles forming materials (mg) (Factor B) Ratio of cholesterol and QAS (Factor C) on **(a)** entrapment efficiency percentage (EE %), **(b)** particle size (PS) and **(c)** zeta potential (ZP).

The entrapment efficiency (EE %) of the prepared quatsomes ranged from 24.4% ± 0.4% to 96.9% ± 0.6% (Table 2). Statistical analysis (p < 0.05) revealed that all examined factors significantly influenced EE %, as illustrated in Figure 1a. Achieving high entrapment efficiency is essential for maximizing drug loading within the vesicular matrix, thereby enhancing therapeutic efficacy, ensuring sustained drug release, and minimizing dosing frequency.

The higher entrapment efficiency (EE %) observed in CPC-based quatsomes compared to CTAB-based ones is primarily related to the structural differences between their quaternary ammonium head groups. CPC contains an aromatic pyridinium ring, while CTAB features an aliphatic trimethylammonium group (Talman et al., 2025; Nemr et al., 2025). The aromatic nature of CPC enhances hydrophobic interactions, π–π stacking, and van der Waals forces within the bilayer, resulting in tighter molecular packing, lower membrane permeability, and superior drug retention. These stronger intermolecular interactions between CPC, cholesterol, and the lipophilic drug collectively produce a more cohesive and stable vesicular structure (Frolov et al., 2025).

In contrast, increasing the total amount of vesicle-forming materials (Factor B) led to a reduction in EE%. When the concentration of cholesterol and quaternary ammonium surfactants exceeded the optimal level, the excess amphiphiles promoted the formation of additional vesicles and micelles. This not only diluted the available drug among a larger number of vesicles but also diverted part of the surfactant to micellar structures that solubilized the drug in the aqueous phase rather than incorporating it into the bilayer (Omolo et al., 2018; Lombardo and Kiselev, 2022).

Furthermore, overly high cholesterol content (Factor C) can disturb the bilayer’s optimal packing arrangement, decreasing its flexibility and limiting drug accommodation within the hydrophobic core. Consequently, both compositional imbalance and phase competition contribute to the observed decline in EE% at higher material concentrations. Also, it could be due to the possible competition between cholesterol and KPN (lipophilic drug) to be incorporated within the lipid membrane (Sun et al., 2010).

#### 3.2.2 Analysis of PS

Regarding PS, PS of the prepared quatsomes ranged from 108.7 ± 2.5 to 328 ± 13.4 nm (Table 2). Statistical analysis of the independent factors revealed that all the independent factors affected the PS of the prepared quatsomes significantly (*p* < 0.05) (Daralnakhla et al., 2021).

Firstly, as demonstrated in Figure 1b, CTAB-based vesicles were larger than those formed with CPC, although both possess identical C16 alkyl chains. This difference could be attributed to variations in head-group structure and counter-ion properties. The flexible, bulky tri-methyl-ammonium head-group in CTAB creates steric hindrance, favouring lower curvature and larger vesicles, whereas the planar, rigid pyridinium ring in CPC allows tighter packing and higher curvature. Additionally, CTAB contains bromide ions, while CPC contains chloride ions. Bromide has a larger atomic radius (∼114 pm) than Cl (∼99 pm), which increases head-group spacing at the vesicle surface. Furthermore, bromide binds more strongly to the positively charged head-groups, reducing the electrostatic repulsion between them. Reduced repulsion allows the bilayer to adopt a lower curvature, producing larger vesicles. In contrast, chloride’s weaker binding maintains higher repulsion, resulting in tighter curvature and smaller vesicles (Kim and Martin, 2023; Helgason et al., 2009).

**FIGURE 2:**
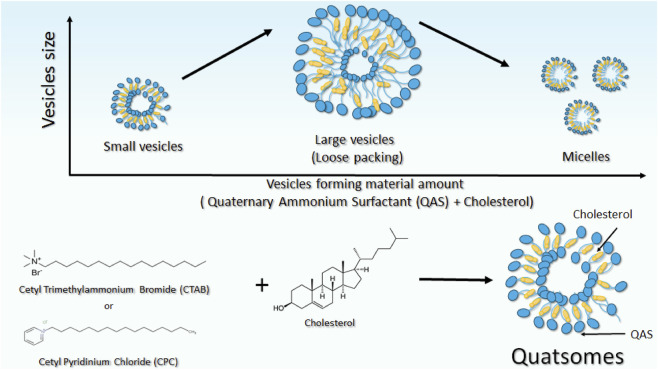
A diagram showing the structure of quatsomes and illustrating the effect of the amount of vesicles forming material on vesicles size.

Regarding factor B, increasing the amount of vesicle-forming materials from 150 mg to 200 mg resulted in a noticeable increase in vesicle size. This behaviour could be attributed to the higher availability of cholesterol and quaternary ammonium surfactant (QAS) molecules, which enhance bilayer assembly and permit looser molecular packing, thereby promoting vesicle growth. However, a further increase to 250 mg led to a decline in particle size (PS), likely due to the preferential formation of micelles at elevated QAS concentrations rather than their incorporation into the vesicular bilayer. Such micellization may deplete surfactant molecules from the vesicle surface, resulting in the formation of more compact structures or fragmented smaller vesicles, as demonstrated in Figure 2. These findings align with previously reported results by Kassem *et al.* (Kassem et al., 2023), confirming that excessive surfactant content can induce structural rearrangement and vesicle size reduction (Omolo et al., 2018; Lombardo and Kiselev, 2022).

With respect to factor C, an increase in the cholesterol-to-QAS ratio was found to correlate with a progressive rise in particle size (PS), which can be explained by a sequence of interrelated structural phenomena. Cholesterol molecules integrate within the bilayer matrix, orienting their hydroxyl groups toward the polar head region while embedding their rigid sterol nuclei among the hydrophobic alkyl chains. This intercalation enhances membrane rigidity and reduces bilayer fluidity, thereby reinforcing vesicular stability (Kaddah et al., 2018; Emad Eldeeb et al., 2019). Concurrently, cholesterol partially disrupts the ordered packing of the lipid components, leading to expansion of the intra-vesicular aqueous domain and facilitating greater water accommodation within the vesicular interior (Adel et al., 2021; Karal et al., 2022). Additionally, cholesterol increased the membrane stiffness, restrict the bilayer’s capacity to achieve high curvature, favouring the formation of larger-diameter vesicles with more stable structural organization (AbouSamra and Salama, 2017).

#### 3.2.3 Analysis of PDI

The polydispersity index (PDI) serves as a critical indicator of the homogeneity and size distribution of nanoscale vesicles, directly influencing their physical stability, reproducibility, and biological performance. Lower PDI values signify a more uniform vesicular population, which is desirable for ensuring consistent and predictable activity. In general, PDI values below 0.5 denote a monodisperse system with acceptable uniformity for pharmaceutical applications (N et al., 2022). In the current study, the PDI values of the optimized KPN-QS ranged between 0.23 ± 0.01 and 0.42 ± 0.03, confirming the formation of vesicles with a narrow and well-controlled particle size distribution across all experimental runs. Such findings reflect the efficiency and reproducibility of the formulation process, suggesting minimal aggregation or vesicle fusion. Furthermore, ANOVA demonstrated that none of the investigated formulation variables significantly affected PDI (*p* > 0.05). This result suggests that the vesicular uniformity was not sensitive to compositional variations within the studied design space. Consequently, although PDI was excluded from the final optimization criteria, the consistently low values demonstrate the robustness of the nanosystem and its appropriateness for localized vaginal drug delivery (Ahmed et al., 2025a).

#### 3.2.4 Analysis of ZP

The zeta potential (ZP) is a critical physicochemical parameter that defines the surface charge density and electrostatic stability of nano-systems. It provides insight into the extent of inter-vesicular repulsion, which directly influences the dispersion uniformity, aggregation tendency, and colloidal stability. Typically, nanocarriers possessing ZP values greater than ±30 mV are considered electrostatically stable, as the strong repulsive forces between charged particles hinder coalescence and sedimentation, thereby ensuring sustained dispersion (El Taweel et al., 2023). In the present study, the developed quatsomal formulations exhibited ZP values ranging from 36.4 ± 0.6 mV to 79.4 ± 3.4 mV, denoting a distinctly high positive surface charge that favours stable colloidal characteristics. Statistical analysis of the independent factors revealed that all the independent factors affected the PS of the prepared quatsomes significantly (*p* < 0.05). Figure 1c illustrates the effect of studied factors.

Regarding factor A, The higher zeta potential observed in Cetyl pyridinium chloride (CPC)–based quatsomes compared with those formulated using Cetyl trimethylammonium bromide (CTAB) can be primarily attributed to intrinsic structural and electrochemical differences between the two surfactants. CPC contains an aromatic pyridinium ring that carries a delocalized positive charge, allowing the charge to be more diffusely distributed and more readily exposed at the vesicle surface. This delocalization enhances electrostatic interactions with the surrounding aqueous medium and contributes to a more pronounced positive surface potential (Talman et al., 2025). In contrast, the quaternary ammonium head group of CTAB features a localized trimethylammonium charge, which, though positively charged, is more shielded by the bulky methyl substituents, resulting in a relatively weaker surface charge manifestation (Taw et al., 2025). Furthermore, the planar geometry of the pyridinium moiety in CPC allows for tighter interfacial alignment and stronger electrostatic repulsion among adjacent head groups, stabilizing the vesicle surface and preventing aggregation. The superior zeta potential exhibited by CPC-based quatsomes, ultimately translating into enhanced colloidal stability and improved mucoadhesive potential (Kim and Martin, 2023; Helgason et al., 2009).

When the total vesicle-forming material increased from 150 mg to 200 mg, a notable reduction in ZP was observed, followed by a subsequent increase when raised further to 250 mg. This biphasic response can be attributed to the complex interplay between electrostatic charge screening, vesicle crowding, and molecular packing density. At lower solid concentrations (150 mg), the vesicles were well dispersed, allowing the cationic head groups of QACs to remain fully ionized and oriented toward the aqueous environment, resulting in pronounced positive surface potential. Increasing the total amount to 200 mg led to a denser dispersion, which promoted partial charge neutralization through increased counter-ion adsorption and inter-vesicular interactions, effectively reducing the measurable ZP. At a higher surfactant concentration (=250 mg), the formation of smaller, more dispersed vesicles with increased surface curvature led to greater exposure of cationic moieties at the aqueous interface, thereby enhancing the electrostatic repulsion between vesicles (Omolo et al., 2018; Lombardo and Kiselev, 2022).

Meanwhile, the cholesterol-to-QAC ratio (factor C) exhibited a positively significant effect on ZP, which may be attributed to cholesterol’s ability to modulate the molecular packing and charge orientation within the bilayer. Cholesterol intercalates between adjacent alkyl chains, aligning its hydroxyl group near the polar head region. This ordered arrangement stabilizes the bilayer and optimizes the orientation of cationic groups, reducing charge shielding and favouring more effective surface charge presentation. At higher cholesterol ratios, the improved bilayer rigidity restricts QAC mobility, minimizing head groups entanglement and promoting enhanced exposure of positively charged sites toward the surrounding aqueous phase. This results in higher measured ZP values and improved colloidal stability.

### 3.3 Optimization of the fabricated quatsomes

The optimization of KPN-QS was performed using Design-Expert^®^ software (Stat-Ease Inc., Minneapolis, United States) to statistically integrate all responses through a desirability approach, which combines multiple criteria into a single numerical value ranging from 0 (undesirable) to 1 (fully desirable) (Helal et al., 2024). The main optimization targets were to maximize entrapment efficiency (EE %) and zeta potential (ZP) while minimizing particle size (PS), ensuring high drug loading, strong colloidal stability, and enhanced mucosal adhesion. Since all formulations exhibited acceptable uniformity, poly-dispersity index (PDI) was excluded from the optimization criteria. The optimized formula achieved a desirability value of 0.840. The optimized formulation was characterized by the use of cetylpyridinium chloride (CPC) as the quaternary ammonium surfactant (factor A), a total vesicle-forming material amount of 150 mg (factor B), and a cholesterol-to-QAC ratio of 1:1 (factor C). This formulation exhibited superior physicochemical characteristics, including EE% of 96.8% ± 0.6%, PS of 113.7 ± 2.32 nm, PDI of 0.35 ± 0.01, and ZP of 72.5 ± 3.3 mV. The close agreement between predicted and experimental results validated the robustness of the model and the accuracy of the software-guided selection (Albash et al., 2021).

### 3.4 *In vitro* analysis of the optimized QS

#### 3.4.1 *In vitro* drug release

**FIGURE 3:**
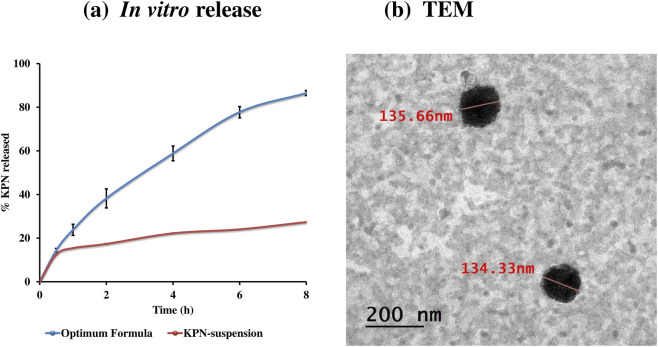
**(a)** *In vitro* release profiles from optimum formula compared to KPN-suspension. **(b)** TEM of the optimum formula.

The *in vitro* release profile of KPN from KPN-OQS and the corresponding KPN-SUSP were evaluated in phosphate buffer saline (pH 4.5, to mimic vaginal acidic nature) containing 5% methanol to maintain sink conditions (Figure 3a) (Ahmed et al., 2025a). KPN-OQ exhibited a biphasic release pattern, characterized by an initial burst release in the first 2 hours (38.21% ± 4.37%), followed by a sustained release phase extending up to 8 h. The rapid initial release may be attributed to the immediate diffusion of the surface-attached or unentrapped drug molecules (Hosny et al., 2018). Subsequently, the release rate slowed, indicating a diffusion-controlled release of quatsomes from the lipid bilayer. At 4 h, the cumulative amount of the drug released reached 58.84% ± 3.39%, and then a plateau-like phase was observed with final release of 86.48% ± 1.17% at 8 h. This sustainment could be attributed to the release of the entrapped drug within the vesicles. In contrast, KPN-SUSP exhibited a significantly slower release profile with only 12.45% ± 0.13% released at 0.5 h, increasing gradually to 27.34% ± 0.03% at 8 h. This slow release is related to the poor aqueous solubility of KPN and the absence of vesicle-mediated transport mechanism in the suspension.

The significantly enhanced release from quatsomes can be attributed to the nanoscale size of the vesicles, which provides a larger surface area for drug diffusion (Ahmed et al., 2025c; Bazaz et al., 2021). In addition, the presence of quaternary ammonium surfactants (QAS) and cholesterol in the bilayer may improve wettability and facilitate drug partitioning into the aqueous medium. Additionally, the positive surface charge of quatsomes could promote electrostatic interactions with the negatively charged buffer components, further enhancing drug release and promote stability (Albash et al., 2025a). Kinetic modeling provided further insight into the release behavior, revealing that the drug release profile of the optimized KPN formulation exhibited the best fit to the Higuchi diffusion model, signifying that diffusion through the vesicular matrix governed the release process.

#### 3.4.2 Transmission electron microscope (TEM)

The TEM micrographs of KPN-OQS showed that the vesicles were distinct with spherical morphology (as shown in Figure 3b). Furthermore, PS measured by Zetasizer was in a good harmony with TEM results (Sayed et al., 2021; Sakr et al., 2023). The vesicles displayed no evidence of aggregation or deformation, indicating the successful incorporation of quaternary ammonium surfactants and cholesterol, which likely contributed to the mechanical stability and integrity of the bilayer structure. The electrostatic repulsion generated by the surface charge effectively prevented vesicle–vesicle aggregation. Furthermore, QAS imparted steric stabilization by forming a hydrated barrier around each nanoparticle, thereby reducing interfacial tension and maintaining colloidal uniformity (Taw et al., 2025; Nemr et al., 2025).

#### 3.4.3 Effect of short term storage stability

KPN-OQS retained its physical characteristics till the end of storage period. A statistical comparison was made between the stored KPN-OQS key physical attributes and those of the freshly prepared samples. PDI (0.41 ± 0.02), EE% (115.21% ± 0.72%), PS (132.51 ± 3.11 nm), ZP (67.25 ± 4.1), and drug release after 8 h (Q8 h: 78.12% ± 0.96%) did not differ significantly (p > 0.05). This is might be attributed to the presence of cholesterol and QAS in its constructs. Cholesterol enhances the packing density of the vesicular membrane and consequently prevents drug leakage during storage (Ahmed et al., 2024b). Furthermore, QAS imparts a strong positive surface charge, providing electrostatic repulsion that inhibits vesicle aggregation. The combined effect of these components contributed to maintaining the vesicle’s integrity and uniformity, thus ensuring the preservation of particle size, surface charge, and entrapment efficiency over the studied period (Taw et al., 2025).

### 3.5 Microbiological and *in vivo* analysis

#### 3.5.1 *In vitro* antifungal activity

KPN demonstrated promising antifungal activity against *C. albicans* ATCC10231 with MIC of 5 ± 0 mg/mL.

#### 3.5.2 *In vivo* fungal vaginal colonization model

**FIGURE 4:**
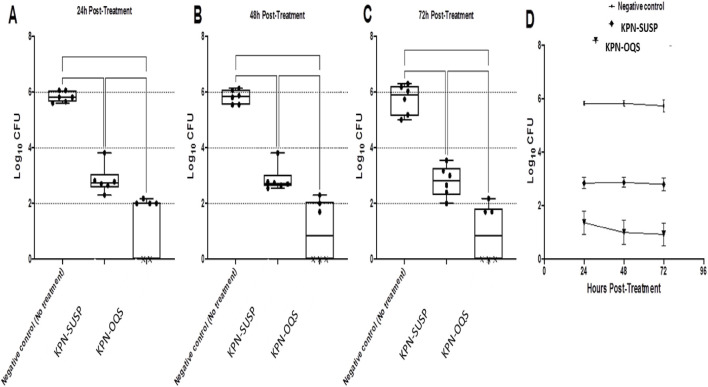
Efficacy of ketoprofen (KPN) loaded quatsomes gel in an *in-vivo* murine model of fungal vaginal infection. Eighteen Wistar rats were divided into three groups (n = 6). One group served as the negative control group and didn’t receive any treatment. The second group was treated with the KPN loaded quatsomes gel and the third group was treated with either the KP loaded gel. Each data point in the figure represents a rat. Results are expressed as box plots of fungal loads recovered from vaginal lumen at 24 h **(A)**, 48 h **(B)** and 72 h **(C)** post-treatment. In addition to, cumulative fungal load at 24, 48 and 72 h **(D)**. The whiskers span the difference between the minimum and maximum readings and the horizontal bar represents the median. and ** indicate that the difference is significant at *p* < 0.01 and < 0.0001, respectively (One-way ANOVA, Tukey’s post-hoc test). X means no colonies were detected in the sample.

A murine model of fungal vaginal infection was used to examine the KPN’s *in vivo* antifungal properties. *C. albicans* ATCC10231 suspension was vaginally injected into three groups of six female Wistar rats. The application of either the KPN-OQS or KPN-SUSP significantly reduced the fungal load recovered from the infected vagina compared to that of the negative control (no treatment) at all tested time intervals (One-way ANOVA, Tukey’s *post hoc* test, p < 0.0001) (Figure 4). The *in vivo* antifungal activity of KPN was considerably increased by the quatsomes, and at all tested time intervals, the antifungal activity of KPN-OQS was significantly higher than that of KPN-SUSP (One-way ANOVA, Tukey’s *post hoc* test, p < 0.01) (Figure 4). The fungal count retrieved from the KPN-OQS on the last day of the experiment was 4.807 and 2.941 logs lower than that of the negative control and KPN-SUSP groups, respectively. Interestingly, by the final day of the trial (72 h after treatment), the KPN-OQS had totally removed the colonized fungus from the vagina of three rats (Figure 4).

#### 3.5.3 Histopathological evaluation

**FIGURE 5:**
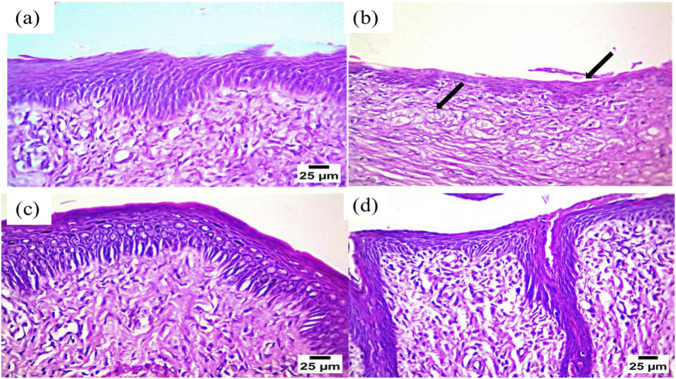
Histopathological study, **(a)** normal control group, **(b)** positive control group, **(c)** KPN-SUSP treated group, and **(d)** KPN-OQS treated group.

As shown in Figure 5, the rat vaginal samples of the normal control group showed normal histological structure of the vaginal wall, including intact stratified epithelial layers and intact lamina propria (a). Positive control (infected samples not treated) showed atrophy of the vaginal epithelium (b). Considering SUSP-treated group (c), photomicrographs showed no marked pathological changes in vaginal epithelium and lamina propria (Hematoxylin and Eosin stain). The KPN-OQS treated group (d) showed organized morphological features of the vaginal wall with intact epithelium without any microscopic alteration. However, histopathological analysis may not detect subtle irritation, mild inflammation, or functional changes. Therefore, future studies are warranted to include *in vitro* cytotoxicity assays on vaginal epithelial cells, evaluation of local inflammatory markers, and monitoring of animal body weight and behavior to provide a more comprehensive assessment of local and systemic toxicity.

## 4 Conclusion

This study describes the design, optimization, and preclinical evaluation of ketoprofen-loaded quatsomes (KPN-QS) as a nanocarrier-based approach for localized vaginal delivery in vulvovaginal candidiasis (VVC). The formulations were developed using quaternary ammonium surfactants and cholesterol via probe sonication and optimized through a 3^1^ × 2^2^ mixed factorial design to achieve favorable physicochemical characteristics, including high entrapment efficiency, nanoscale particle size, and positive surface charge.

The optimized KPN-QS exhibited uniform nanosized vesicles with sustained drug release and satisfactory storage stability. *In vitro* and *in vivo* microbiological evaluations demonstrated enhanced antifungal activity of KPN-QS compared with the corresponding drug suspension, supporting the potential advantage of nanocarrier-mediated delivery. Histopathological examination indicated preservation of vaginal mucosal integrity, suggesting acceptable local tolerability under the tested conditions.

Taken together, these findings support the feasibility of quatsomes as a nanocarrier platform for repurposed ketoprofen delivery and provide proof-of-concept evidence for their potential utility in VVC management. However, given the preclinical nature of the study and the absence of comparisons with standard antifungal therapies and comprehensive safety assessments, further investigations are required. Future studies should focus on detailed toxicity profiling, evaluation of effects on vaginal microbiota, mechanistic validation, and comparative efficacy against established antifungal agents before clinical translation can be considered.

## Data Availability

The raw data supporting the conclusions of this article will be made available by the authors, without undue reservation.
